# Adult-Onset Still’s Disease Causing Aortitis Diagnosed Through the Investigation of Inflammatory Anemia in a Chronic Pseudogout Patient

**DOI:** 10.7759/cureus.78712

**Published:** 2025-02-07

**Authors:** Ryosuke Kashiwaba, Kohei Oka, Ryuichi Ohta

**Affiliations:** 1 Family Medicine, International University of Health and Welfare Graduate School of Health Sciences, Tokyo, JPN; 2 Community Care, Unnan City Hospital, Unnan, JPN

**Keywords:** adult-onset still's disease, anemia, aortitis, calcium pyrophosphate deposition disease, chronic disease, family medicine, general medicine, inflammatory cytokines, interleukin-6 receptor antagonists

## Abstract

Adult-onset Still’s disease (AOSD) is a rare systemic autoinflammatory disorder that can present with fever, arthritis, and systemic inflammation, often complicating underlying chronic conditions. This report describes the case of a 71-year-old male patient with chronic kidney disease-related renal anemia and pseudogout (calcium pyrophosphate deposition disease (CPPD)) treated on an outpatient basis. The patient presented to our hospital with rectal bleeding lasting for two to three weeks, leading to the identification of rapidly progressing anemia through blood tests. He was hospitalized for further investigation of acute anemia and received a packed red blood cell transfusion. However, no definitive findings were obtained, and he was discharged with symptomatic treatment. Subsequently, the patient developed abdominal pain and aortic wall thickening, leading to hospitalization at a higher-level hospital. No definitive diagnosis was made, and the symptoms improved spontaneously. It was initially suspected to be inflammation due to pseudogout. While continuing outpatient treatment at our hospital, the patient developed elevated liver enzymes, increased neutrophil counts, elevated ferritin, and polyarthritis. Given the constellation of findings, AOSD was diagnosed, which was likely the underlying driver of inflammation and anemia in this case. Treatment with prednisolone and an interleukin-6 inhibitor led to symptom relief and improvement in blood test results. This case highlights the diagnostic challenges of AOSD, particularly its rare onset in elderly patients and its potential to mimic other chronic inflammatory conditions such as pseudogout. It also underscores the importance of considering AOSD in elderly patients with acute anemia and the need for accurate and prompt treatment.

## Introduction

Anemia of chronic disease (ACD), also known as anemia of inflammation, is a common type of anemia associated with chronic conditions such as infections, autoimmune diseases, and malignancies [1]. It is the second most common cause of anemia globally and is primarily driven by the dysregulation of iron metabolism and erythropoiesis due to persistent inflammation. Inflammatory cytokines, including interleukin-6 (IL-6) and tumor necrosis factor-alpha (TNF-α), promote hepcidin overproduction, which inhibits iron absorption and traps iron in macrophages, ultimately impairing red blood cell production [2,3].

ACD affects a significant proportion of elderly individuals, hospitalized patients, and those with chronic inflammatory diseases. Studies estimate that 12% of community-dwelling elderly individuals, 40% of hospitalized patients, and 47% of nursing home residents have anemia, with many cases attributed to chronic inflammation [4]. Additionally, ACD is present in approximately 60% of patients with rheumatoid arthritis and is frequently observed in inflammatory bowel diseases such as ulcerative colitis and Crohn’s disease [5,6].

Diagnosing ACD can be challenging, as it often overlaps with other forms of anemia, such as iron deficiency anemia or anemia related to chronic kidney disease. The condition is characterized by normocytic or microcytic anemia with low serum iron levels despite normal or increased ferritin [7]. However, its presentation can be nonspecific, leading to misdiagnosis, particularly in patients with multiple comorbidities. Given this diagnostic complexity, it is crucial to reassess cases where anemia persists or worsens despite initial treatment.

We report a case of a 71-year-old patient with chronic kidney disease and pseudogout, initially treated for ACD, who developed severe anemia, polyarthritis, and systemic inflammation. Further evaluation led to the unexpected diagnosis of adult-onset Still’s disease (AOSD) with aortitis, a rare presentation in elderly patients. This case highlights the diagnostic challenges of inflammatory anemia in older adults and underscores the importance of considering systemic autoinflammatory diseases in patients with unexplained anemia and persistent inflammation.

## Case presentation

A 71-year-old male patient receiving treatment for chronic inflammatory anemia at our hospital presented with acute anemia progression and was referred to the general medicine department. He had been treated for normocytic anemia due to chronic kidney disease-related anemia for one year. Approximately two to three weeks before presentation, he noticed minor rectal bleeding from hemorrhoids, leading to the primary care clinic where severe anemia (hemoglobin (Hb) 6.7 g/dL) was detected, prompting referral to our hospital. Emergent gastrointestinal endoscopy revealed no significant bleeding source. He was admitted for further evaluation with neck-to-pelvic computed tomography (CT) and colonoscopy, which did not detect any causes of anemia. He was transfused with two units of packed red blood cells, leading to symptomatic improvement, and was discharged for observation in the outpatient department. Hb remained around 8 g/dL, raising concerns about pseudogout and renal anemia as the underlying cause. 

**Figure 1:**
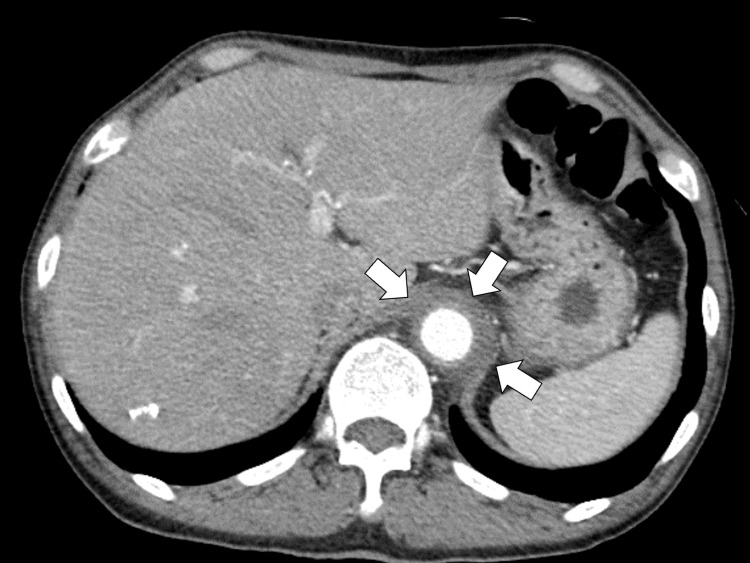
Contrast-enhanced abdominal computed tomography showing the wall thickening of the descending aorta (white arrows)

One week later, he developed acute abdominal pain with elevated inflammatory markers and aortic wall thickening on contrast-enhanced CT, suggesting aortic dissection or aortitis, and was transferred to a tertiary hospital (Figure 1).

Despite further investigations in the tertiary hospital, including positron emission tomography (PET), no definitive diagnosis was made, and symptoms improved with one week of intravenous methylprednisolone of 40 mg attributed to systemic inflammation from pseudogout. 

His past medical history included hypertension, colonic polypectomy, bilateral knee arthritis, pseudogout, chronic kidney disease, chronic cholestatic liver disease, hyperuricemia, and lumbar spinal stenosis. His medications included febuxostat 10 mg, rosuvastatin 2.5 mg, enalapril maleate 10 mg, potassium binder 75 g, lubiprostone 4.8 μg, prednisolone 10 mg, and linaclotide 0.5 mg.

On presentation, his vital signs were as follows: clear consciousness, body temperature 39.6°C, blood pressure 140/65 mmHg, pulse rate 76 bpm, oxygen saturation (SpO2) 96% on room air, and respiratory rate 18/minute. Physical examination revealed swelling and tenderness in the wrist and knee joints without other remarkable findings on the chest, abdomen, or back. Blood tests showed neutrophil-dominant leukocytosis, elevated liver enzymes, moderate anemia, hyperferritinemia, and increased C-reactive protein (CRP) and erythrocyte sedimentation rate (ESR) levels (Table 1).

**Table 1:** Initial laboratory data of the patient UIBC: unsaturated iron-binding capacity; Na: sodium; K: potassium; Cl: chloride

Parameters	Patient values	Reference ranges
White blood cells	10.4	3.5–9.1 × 10^3^/μL
Neutrophils	90.4	44.0–72.0%
Lymphocytes	9	18.0–59.0%
Hemoglobin	7.9	11.3–15.2 g/dL
Hematocrit	24.2	33.4–44.9%
Mean corpuscular volume	84.3	79.0–100.0 fl
Red blood cell distribution width	18.5	11.5~14.5%
Platelets	26	13.0–36.9 × 10^4^/μL
Albumin	2.2	3.8–5.3 g/dL
Total bilirubin	3.4	0.2–1.2 mg/dL
Aspartate aminotransferase	38	8–38 IU/L
Alanine aminotransferase	40	4–43 IU/L
Lactate dehydrogenase	275	121–245 U/L
Erythrocyte sedimentation rate	110	2~10mm
Blood urea nitrogen	20.7	8–20 mg/dL
Creatinine	1.13	0.40–1.10 mg/dL
Serum Na	139	135–150 mEq/L
Serum K	2.5	3.5–5.3 mEq/L
Serum Cl	103	98–110 mEq/L
C-reactive protein	14.2	<0.03 mg/dL
Erythrocyte sedimentation rate	65	<15 mm/hour
Ferritin	844.9	14.4–303.7 ng/mL
Total homocysteine	10	7.0~17.8 nmol
Erythropoietin	29.7	~29.0mIU/mL
Haptoglobin	211	mg/dL
Cyclic citrullinated peptide	<0.6	~5U/mL
Rheumatoid factors	1	0～15 IU/mL
Serum iron	13	54~181μg/dL
UIBC	163	111~255μg/dL
Iron binding capacity	176	μg/dL
Iron saturation	7.4	%
Reticulocytes	3.4	0.2~2.7%
Reticulocytes production index	0.71	1.99~2.99
Zinc	65.5	80~130μg/dL
Thyroid stimulation hormone	4.60	0.35~4.94
Free T4	1.1	0.70~1.48ng/dL
Serum copper	210	66~130μg/dL
Vitamin 12	497	187~883μg/dL
Folic acid	5.4	ng/dL
Iron saturation	7.4	%
Urine test	-	-
Leukocyte	Negative	Negative
Protein	Negative	Negative
Blood	Negative	Negative

**Figure 2:**
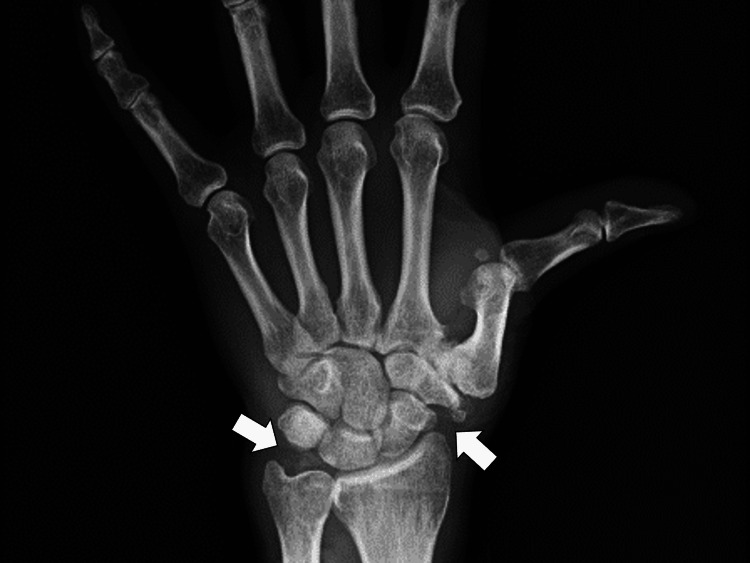
X-ray showing calcified deposits in the left wrist joint

Magnetic resonance imaging (MRI) of the bilateral wrists, knees, and spine, and upper and lower gastrointestinal endoscopy for anemia investigation was performed. X-ray showed calcified deposits in the wrist joints (Figure 2).

No significant findings were observed on spinal MRI or endoscopy. Despite another transfusion of two units of packed red blood cells, Hb levels continued to decline, and inflammatory markers remained elevated with a CRP of 12.3 mg/dL.

Although no rash was observed, persistently elevated liver enzymes, neutrophil counts, ferritin, and polyarthritis led to a clinical diagnosis of AOSD based on Yamaguchi diagnostic criteria [6]. We confirmed that AOSD could trigger aortitis in hyperinflammatory conditions. The patient was treated with prednisolone (30 mg) and intravenous tocilizumab (400 mg). His inflammatory markers and anemia improved rapidly following treatment. He was discharged on day 10 of the admission. In the outpatient department, his prednisolone was tapered biweekly, and tocilizumab was administered monthly.

## Discussion

This case highlights the diagnostic complexity of AOSD, particularly in the context of late-onset presentation and overlapping comorbid conditions [7]. AOSD is a rare systemic autoinflammatory disorder with hallmark features of fever, arthritis, and an evanescent salmon-colored rash, often accompanied by leukocytosis, hyperferritinemia, and systemic inflammation. While anemia due to chronic inflammation is a known complication, acute anemia as a prominent presentation is uncommon and underscores the atypical nature of this case.

AOSD predominantly affects younger adults, with a peak incidence in the third to fifth decades of life. Late-onset AOSD, defined as onset after the age of 60, is exceedingly rare and can present with atypical features [7,8], as demonstrated in this 71-year-old patient. Late-onset cases are often underrecognized due to their overlap with other systemic inflammatory diseases and age-related comorbidities [9,10]. In this case, CKD and pseudogout further obscured the diagnosis, as both are independently associated with anemia and systemic inflammation. AOSD should be included in the differential diagnosis of a patient with hyperinflammatory conditions.

Notably, while widely used for diagnosing AOSD, the Yamaguchi criteria require excluding other inflammatory, infectious, and neoplastic conditions [11]. In elderly patients, this process becomes particularly challenging due to the higher prevalence of comorbidities and atypical disease presentations [12]. This diagnostic complexity is well-documented in the literature, with studies emphasizing the need for high clinical suspicion and comprehensive diagnostic workups in older populations presenting with systemic inflammation [12]. In older patients, AOSD can be caused by the interaction among multiple acute and chronic diseases, so general physicians should include AOSD in the list of differential diagnosis list with unknown chronic inflammatory conditions [13].

ACD is a multifactorial condition driven by the dysregulated production of inflammatory cytokines such as IL-6, TNF-α, and interferon-gamma (IFN-γ) [8]. These cytokines promote the overproduction of hepcidin, a peptide hormone that regulates iron homeostasis by inhibiting intestinal iron absorption and trapping iron in macrophages. Hepcidin-mediated iron dysregulation leads to functional iron deficiency and impaired erythropoiesis [14].

In the context of AOSD, IL-6 plays a central role in driving systemic inflammation and anemia. In this case, the efficacy of tocilizumab, an IL-6 receptor inhibitor, underscores the critical contribution of IL-6 to the patient's inflammatory and hematologic abnormalities. Rapid Hb levels and systemic inflammation improvement following tocilizumab therapy align with previous reports highlighting the effectiveness of targeting IL-6 in AOSD-related anemia [15,16]. Diagnostic treatment with tocilizumab can be challenging and with a high threshold. Still, as this case shows, severe anemia and hyperinflammatory conditions ruling out critical diseases, such as bacterial infection, can drive the usage of tocilizumab for AOSD without any rash.

Another noteworthy aspect of this case is the development of vasculitis, a rare but recognized complication of AOSD. Systemic vasculitis in AOSD is thought to result from hyperinflammatory states driven by cytokine overproduction [17]. Vasculitis is uncommon in late-onset AOSD, with only a handful of cases reported in the literature [18]. Early recognition and treatment of such vascular complications are critical, as they can lead to severe outcomes such as aortic aneurysm or dissection [18,19]. This case reinforces the need for clinicians to maintain a high index of suspicion for vascular involvement in patients with AOSD who present with atypical symptoms such as abdominal pain or imaging findings of aortic wall thickening. MRI or PET-CT imaging can provide valuable diagnostic insights in such scenarios. General physicians should consider AOSD in the differential diagnosis of aortitis with unknown inflammatory conditions.

The global prevalence of AOSD varies, with higher reported incidences in Asian populations compared to Western countries [7]. This geographical variation may reflect genetic, environmental, or diagnostic factors. In Japan, where this case was reported, the late-onset presentation of AOSD may be increasingly relevant, given the aging population and the rising prevalence of age-related comorbidities [7]. This case underscores the importance of interdisciplinary collaboration in managing complex presentations of systemic inflammatory diseases. The involvement of general physicians, rheumatologists, and hematologists was pivotal in this patient's diagnostic and therapeutic journey [20]. Furthermore, it highlights the potential of biological therapies, such as IL-6 inhibitors, in providing targeted and effective treatment for refractory cases of AOSD.

## Conclusions

This case underscores the diagnostic complexities of AOSD in elderly patients, particularly with overlapping conditions like chronic kidney disease and pseudogout. The rare late-onset presentation and development of complications such as aortitis highlight the importance of maintaining high clinical suspicion and conducting thorough evaluations. The successful use of IL-6 inhibitor therapy emphasizes the role of cytokines in chronic disease anemia and systemic inflammation in AOSD. Clinicians should consider AOSD in elderly patients with unexplained anemia and systemic inflammation, as early diagnosis and targeted therapy are crucial for achieving optimal outcomes and preventing severe complications.
